# Supplementary material for: Study of the Effects of the Structure of Phthalazinone’s Side-Group on the Properties of the Poly(phthalazinone ether ketone)s Resins
Source: Polymers (Basel). 2019 May 5;11(5):803. doi: 10.3390/polym11050803 (PMC6571746; doi:10.3390/polym11050803)
Supplement: Supplementary file 1 [file polymers-11-00803-s001.pdf]

polymers

MDPI

Supplementary data for

Study of the Effects of the Structure of Phthalazinone's Side-group on the Properties of the Poly(phthalazinone ether ketone)s Resins

Feng Bao [1,2], Fengfeng Zhang [1,2], Chenghao Wang [1,2], Yuanyuan Song [3], Nan Li [1,2], Jinyan Wang [1,2*], Xigao Jian [1,2]

[a] State Key Laboratory of Fine Chemicals Dalian University of Technology, Dalian 116024, China;
[b] Department of Polymer Science and Materials, Dalian University of Technology, Dalian 116024, China;
[c] Shenzhen China Start Optoelectronic Technology Co., Ltd, Shenzhen 518132, China.
bfisvip@163.com (F.B.); Zhangfeng0908@126.com (F.Z.); wangchh@mail.dlut.edu.cn (C.W.); yysong1211@163.com (Y.S.); polymerlinan@dlut.edu.cn (N.L); jian4616@dlut.edu.cn (X.J.);

* Correspondence: wangjinyan@dlut.edu.cn (J.W.);
Tel.: +86-411-8498-6092 (J.W.);

This supporting information is composed of the total of 7 pages, including 7 Figures

Page 2-5:

Figure S1. 1H-NMR and 13C-NMR patterns of the prepared monomers, (a) is 1H-NMR and (b) is 13C-NMR.

Figure S2. 2D NMR spectrum of DHPZ-M and DHPZ-Ph: (a) is HMBC of DHPZ-M, (b) is HMBC of DHPZ-Ph, (c) is HSQC of DHPZ-M and (d) is HSQC of DHPZ-Ph

Figure S3. The HRMs spectrum of DHPZ-M and DHPZ-Ph: (a) is DHPZ-M and (b) is DHPZ-Ph.

Figure S4. FTIR pattern of the DHPZ-M

Figure S5. The GPC traces of PPEK, PPEK-M and PPEK-Ph.

Figure S6. The WAXD patterns of PPEKs resins.

Figure S7. The stress-strain curves of the resin (PPEK, PPEK-M and PPEK-Ph).

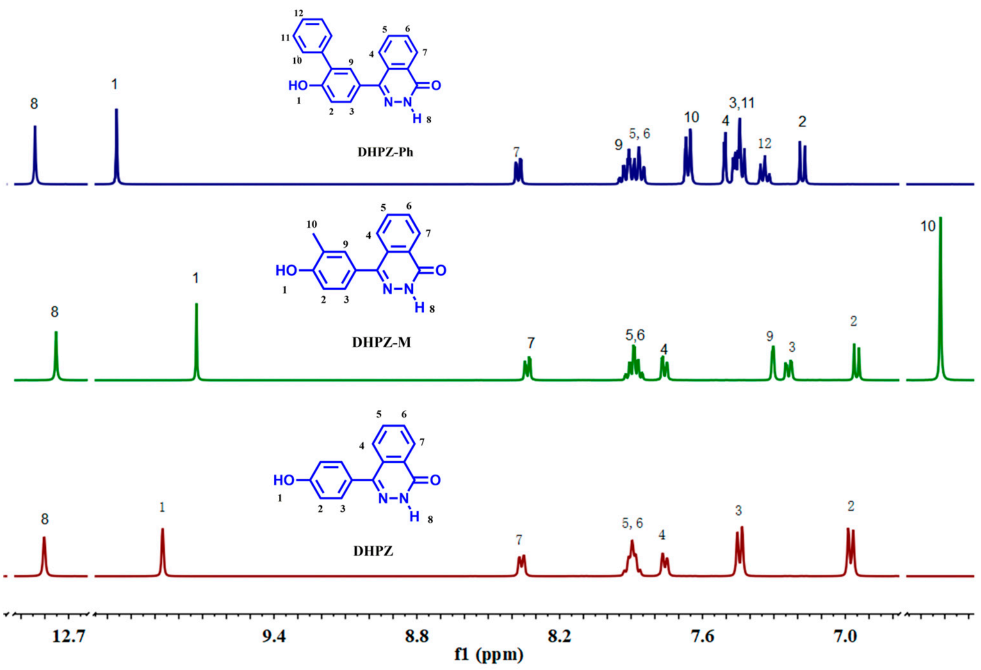

(a)

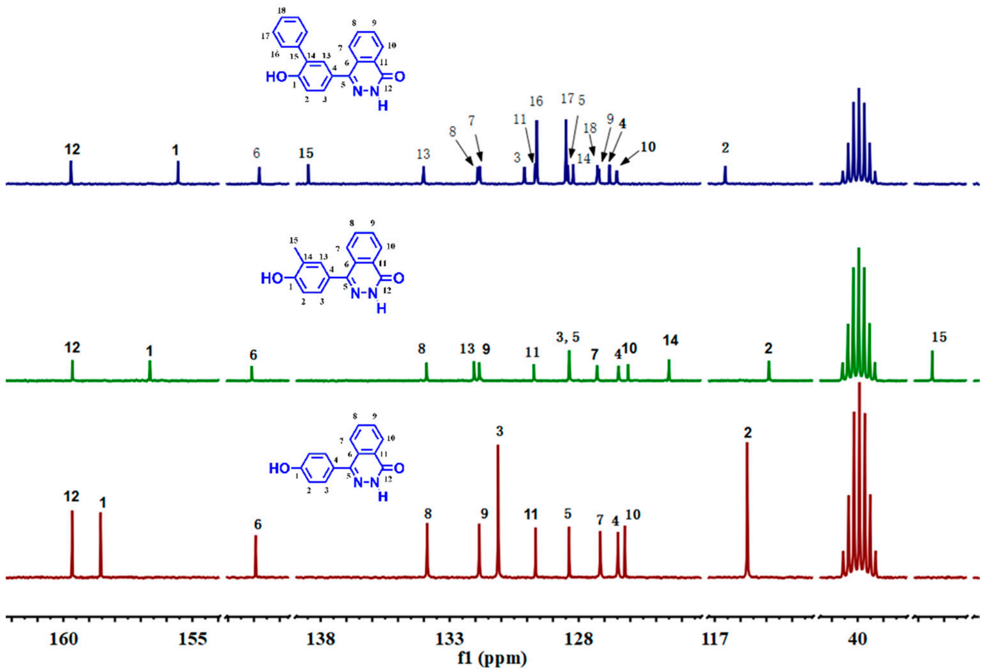

(b)

Figure S1. 1H-NMR and 13C-NMR patterns of the prepared monomers, (a) is 1H-NMR and (b) is 13C-NMR.

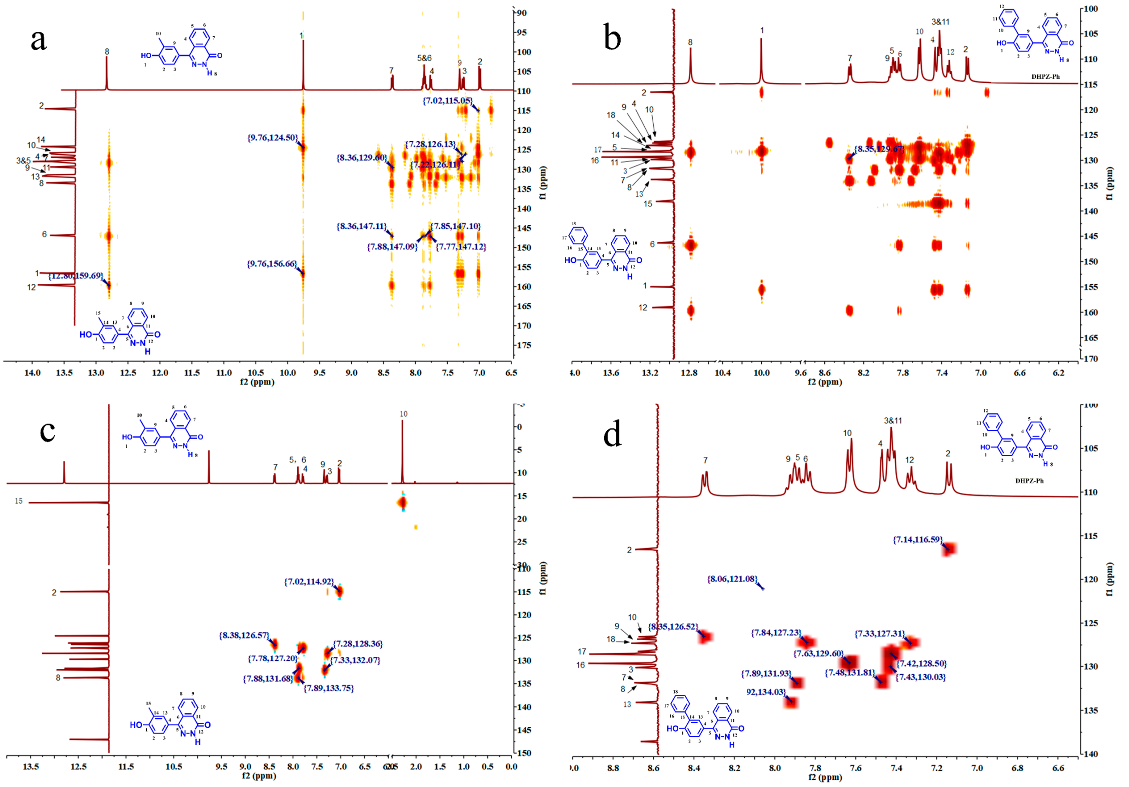

Figure S2. 2D NMR spectrum of DHPZ-M and DHPZ-Ph: (a) is HMBC of DHPZ-M, (b) is HMBC of DHPZ-Ph, (c) is HSQC of DHPZ-M and (d) is HSQC of DHPZ-Ph.

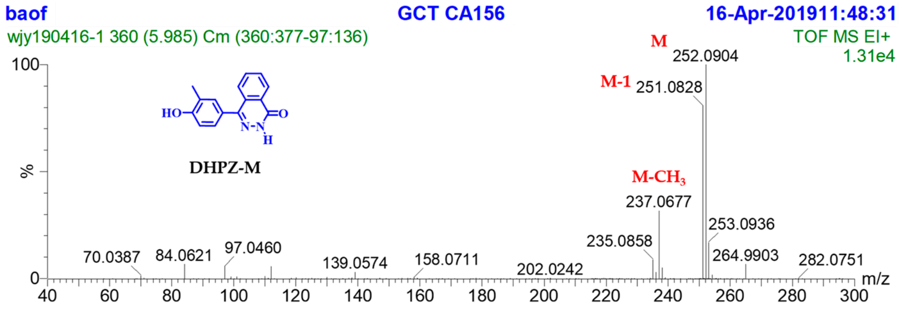

(a)

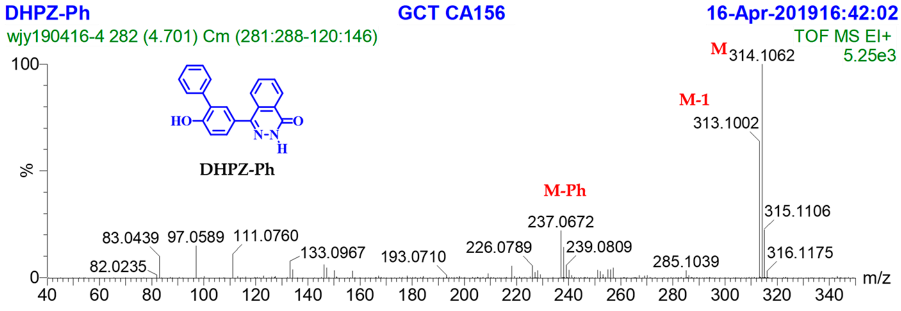

(b)

Figure S3. The HRMs spectrum of DHPZ-M and DHPZ-Ph: (a) is DHPZ-M and (b) is DHPZ-Ph.

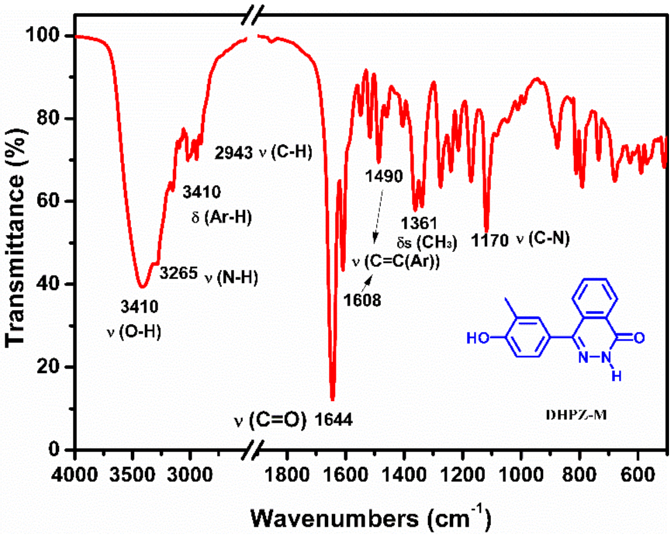

Figure S4. FTIR pattern of the DHPZ-M

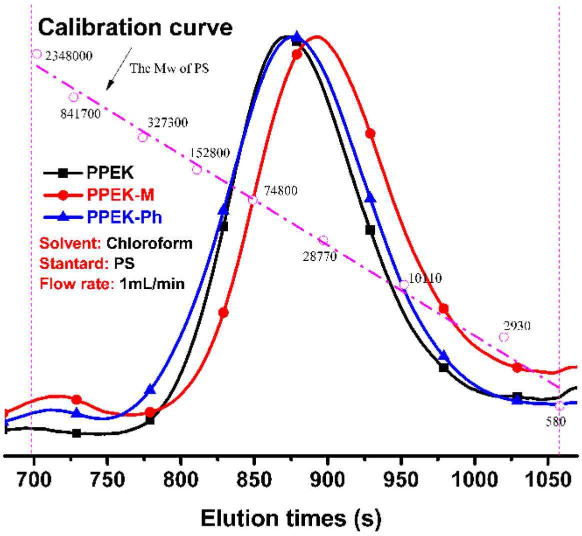

Figure S5. The GPC traces of PPEK, PPEK-M and PPEK-Ph.

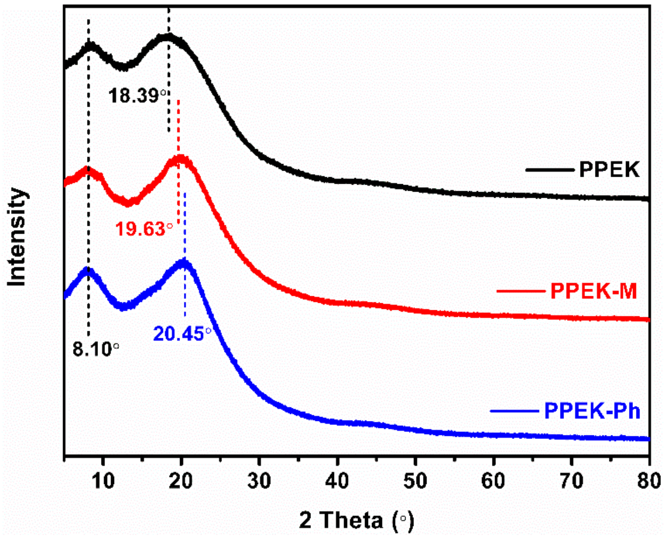

Figure S6. The WAXD patterns of PPEKs resins.

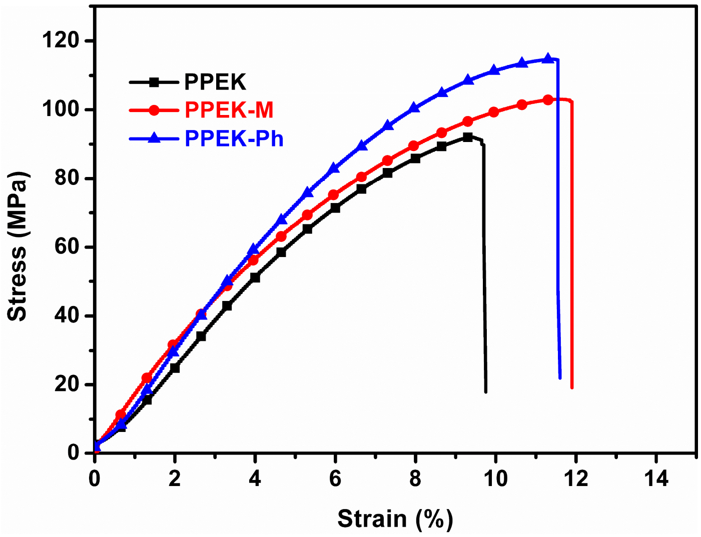

Figure S7. The stress-strain curves of the resin (PPEK, PPEK-M and PPEK-Ph).
